# Institutional Barriers, Educational Decisions, and the Forging of Migrant Social Identity: Evidence from China’s Cross-Regional Enrollment Policies

**DOI:** 10.3390/bs16091488

**Published:** 2026-08-26

**Authors:** Chen Li, Qiao Wen, Xu Li

**Affiliations:** 1 Business School, Beijing Normal University, Beijing 100875, China; lichenn@mail.bnu.edu.cn; 2 Education Development Research Center, Beijing Academy of Educational Sciences, Beijing 100036, China

**Keywords:** migrant children, social identity formation, structural constraints, educational inequality

## Abstract

The social identity of migrant children is not merely a demographic label but is actively constructed and constrained by institutional structures. In China, a rapidly growing migrant population faces stringent institutional barriers, namely cross-regional enrollment policies with restrictive eligibility conditions, which exclude migrant children from local high school education. This study investigates how these structural factors shape the educational decisions and, consequently, the social identity formation of migrant children. Using data from the China Migrants Dynamic Survey and a cohort-based difference-in-differences (DID) approach, we employ an innovative strategy to identify students’ high school enrollment locations by leveraging information on migration timing and birth cohorts. Our findings reveal that strict institutional barriers significantly reduce the likelihood of high school attendance in destination areas, forcing families into a critical trade-off between children’s educational opportunities and family co-residence, which serves as a key site where migrant identity is negotiated. Residence requirements and school-type restrictions emerge as the most binding constraints. Importantly, these policies reinforce the role of household economic capital in accessing education while only partially mitigating disparities linked to cultural capital, suggesting a stratified reproduction of migrant social identities. Mechanism analysis indicates that early co-migration of children serves as a key behavioral channel, illustrating how structural barriers reshape family strategies. Ultimately, this study demonstrates that the educational decisions of migrant families are strongly constrained by institutional arrangements, which in turn shape the salience, meaning, and developmental trajectory of their migrant social identity. Reducing access thresholds and providing more flexible pathways are essential for fostering more equitable identity outcomes.

## 1. Introduction

As a crucial component of the labor market, the floating population plays an indispensable role in promoting urban economic growth and structural transformation (Chan, 2010). From the perspective of social sustainability, ensuring access to basic public services for their family members is a fundamental prerequisite for achieving inclusive urbanization. Beyond material welfare, access to these services is also central to the construction of social belonging and the formation of social identities among migrant families in destination cities. However, against the backdrop of regional development disparities and population agglomeration, local governments in destination areas often adopt institutional arrangements to selectively allocate public resources (Hung, 2022), among which the access mechanisms governing educational resources are especially salient (Xu & Wu, 2022).

Within China’s current institutional framework, the household registration (hukou) system continues to shape, to a certain extent, not only the distribution of public services but also the boundaries of social membership between migrant and local populations. In this sense, institutional arrangements do not merely regulate access to education but also embed structural signals about who belongs, who is included, and under what conditions such inclusion is possible. To mitigate the educational exclusion faced by migrant children, the government has progressively advanced reforms in the education system (Y. Chen et al., 2019). Since the early 21st century, the policy framework centered on the principles of “two priorities” and “two inclusions” has largely addressed access to compulsory education, enabling migrant children to receive basic education in destination areas (Bo & Wang, 2025). This phase of institutional evolution reflects a broader shift toward greater inclusiveness, while still maintaining differentiated boundaries of educational membership across social groups.

As the demand for migrant children to continue their education in destination areas after completing compulsory education increased (Goodburn, 2020), the Chinese government introduced a national document in 2012 aimed at ensuring equal access to further education (General Office of the State Council, 2012). This document emphasized the need to guarantee migrant children’s rights to education and opportunities for advancement. Following this initiative, local governments across China implemented specific measures allowing migrant children to participate in entrance examinations in destination areas after completing compulsory education, hereafter referred to as cross-regional enrollment policies. These policies formally recognize migrant children’s right to access upper-secondary education in destination areas and provide an institutional foundation for expanding their educational participation beyond compulsory schooling.

Nevertheless, compared with compulsory education, access to post-compulsory education is considerably more complex (Deng & Gao, 2024). As highly selective mechanisms, high school and college entrance examinations are characterized by the competitive allocation of educational opportunities (Ma & Wang, 2025). Given substantial regional disparities in educational resources, a full liberalization of cross-regional advancement opportunities could induce excessive population concentration in areas with superior educational resources, thereby undermining the capacity and stability of local education systems (Liu et al., 2025). Consequently, local governments have generally introduced conditional access requirements—such as social insurance contributions, proof of stable housing, and continuous employment—thus forming a governance model of “conditional openness”. These conditions not only regulate mobility but also implicitly define the boundaries of legitimate membership in the urban education system, thereby shaping the conditional nature of migrant families’ social belonging.

While such access mechanisms may help maintain the relative stability of educational resource allocation in the short term, their long-term implications for educational equity and the formation of migrant social identities remain insufficiently examined. In particular, in regions with high entry thresholds, some migrant children may be compelled to choose between migrating with their parents and returning to their places of origin for schooling. This trade-off not only shapes their educational trajectories but also produces differentiated experiences of belonging, which may exert lasting effects on their psychological development (Zheng et al., 2022) and human capital accumulation (C. Wang & Xu, 2025). Accordingly, how to optimize institutional design under resource constraints, so as to balance educational opportunity equity with rational population mobility and more inclusive identity outcomes, constitutes a critical question.

Building on this concern, this study adopts an integrated perspective of social sustainability, educational equity, and social identity formation, and proceeds along four analytical dimensions. First, at the level of policy effects, this study examines whether the stringency of access thresholds weakens the effectiveness of cross-regional school advancement policies and reduces the likelihood that migrant children attend high school in destination areas. This question is central to assessing whether institutional reforms achieve both educational inclusion and identity inclusion. Second, at the level of institutional design, this study differentiates among various types of access conditions (e.g., social insurance contributions, housing requirements, and employment stability) and analyzes their heterogeneous constraining effects, with the aim of identifying the key institutional factors shaping access to educational opportunities and boundaries of social membership. Third, given that access thresholds are closely tied to family resource endowments, their implementation may unintentionally reinforce pre-existing inequalities, thereby shaping stratified patterns of educational access and differentiated identity formation. Therefore, from the perspective of equity and identity, this study investigates the heterogeneous impacts of institutional constraints across families with different socioeconomic backgrounds. Fourth, this study investigates whether cross-regional enrollment policies increase the likelihood that children accompany their family during both compulsory and post-compulsory education stages, thereby influencing the spatial context in which social identity is formed and negotiated.

This study makes three main innovations. First, in terms of the research subject, most existing studies focus on migrant children who have already moved with their parents (Y. Chen et al., 2019), paying insufficient attention to potential sample selection bias arising from the “left behind versus migrant” dichotomy. Children who remain in or return to their place of origin are also part of the broader migrant population, yet their information is typically unobserved. Moreover, migration decisions are not random but are influenced by the stringency of cross-regional enrollment policies (B. Chen et al., 2026). Focusing solely on migrant children may therefore lead to biased estimates. By jointly considering migrant and left-behind children, this study mitigates sample selection concerns and provides a more accurate assessment of policy effects. In this study, ‘children from migrant families’ refers to the overall sample, while ‘migrant children’ specifically denotes those who migrated with their families, and ‘left-behind children’ refers to those who remained in their place of origin. Second, regarding outcome variables, the existing literature primarily examines whether children attend high school, with limited attention to where they attend. However, the fundamental objective of cross-regional enrollment policies is not only educational access but also the spatial incorporation of migrant children into destination education systems, which is closely linked to their sense of belonging and identity formation. Although some studies consider both migrant and left-behind children and explore the impact of such policies on high school attendance and residential status, they are unable to identify the actual location of schooling (Y. Y. Chen et al., 2023). This study addresses this gap by explicitly identifying whether migrant children attend high school in destination areas, thereby providing a more policy-relevant and identity-sensitive evaluation. Third, in terms of policy measurement, prior studies often rely on composite indices to capture policy stringency, with limited attention to the specific requirements outlined in policy documents (Zou et al., 2023). Different types of access conditions, such as social insurance contributions, proof of stable employment, and legal housing, may impose heterogeneous constraints on migrant families while simultaneously signaling differential levels of institutional acceptance. By returning to the original policy texts and constructing disaggregated measures based on specific requirements, this study examines the distinct effects of different access conditions, offering more targeted implications for both policy design and the understanding of structurally conditioned social identity formation.

## 2. Literature Review

### 2.1. Migrant Children’s Education: An International Perspective

International research on migrant children’s education increasingly emphasizes that educational inequality arises from multiple interacting factors rather than a single source (OECD, 2018; Portes & Zhou, 1993). From a psychological perspective, these multi-level disparities are heavily mediated by individual acculturation processes, mental health challenges, and psychological resilience (Berry, 1997; Motti-Stefanidi, 2018). Existing studies show that educational and developmental outcomes are jointly shaped by structural conditions, individual backgrounds (such as psychological coping resources), and school environments (Pong & Hao, 2007; Schnepf, 2007).

One key perspective focuses on the role of local context. Research by Olof Åslund finds that migrant students benefit from living in communities with higher levels of educational resources, particularly when co-ethnic networks provide access to information and support (Åslund et al., 2011). This suggests that social environment plays an important role in facilitating academic success. Another strand highlights differences across migrant groups. Evidence from large-scale assessments shows that students’ prior educational experiences and country of origin significantly influence achievement. Migrants from stronger education systems tend to experience smaller performance gaps, indicating that pre-migration resources continue to matter after relocation (Giannelli & Rapallini, 2016).

Barriers to educational access remain a central concern. Institutional constraints, including legal status and policy design, can restrict opportunities and contribute to unequal outcomes (Dryden-Peterson, 2016). Studies further show that limited access to education may coexist with labor market disadvantage, which together constrain long-term mobility (Baker et al., 2023). At the same time, critical research points out that migrant students are often framed as disadvantaged in educational discourse (Brunner et al., 2024). Scholars argue that such representations may shape expectations and reinforce inequality within institutions (Lukala & Dervin, 2026). This perspective highlights the importance of examining how policies and practices are constructed. Some studies also find that second-generation migrants demonstrate strong educational motivation and upward mobility (Gomensoro et al., 2025), suggesting that disadvantages can be mitigated under certain conditions. At the school level, language proficiency emerges as a major challenge affecting both learning and social interaction (Cummins, 2000). However, support from teachers and peers can help students adapt and improve their performance (Kaukko et al., 2022).

Overall, the literature indicates that migrant children’s education is shaped by context-dependent and layered mechanisms. A comprehensive understanding requires attention to structural factors, individual resources, and institutional practices.

### 2.2. Institutional Design and Regional Variation

China’s cross-regional enrollment policies have gradually evolved from expanding access to compulsory education to regulating educational opportunities at the senior secondary and tertiary levels. Y. Chen et al. (2019) reviewed the evolution of migrant children’s education policies and argued that although national reforms have gradually improved educational access, substantial disparities persist because local governments retain considerable discretion in determining admission requirements. These locally defined eligibility rules have become an important source of unequal educational opportunities for migrant children.

Building on this perspective, Y. Wang and Sercombe (2023) examined the design and implementation of education policies for migrant children and found considerable regional variation in policy content and admission requirements. Their study demonstrated that local governments adopted different admission requirements, including residence certificates, employment records, and other documentation requirements, due to variations in local implementation conditions and governance constraints. These findings provide an important foundation for understanding the institutional characteristics of cross-regional enrollment policies.

### 2.3. Policy Effects on Educational Access and Migration Decisions

The existing literature examines the effects of cross-regional enrollment policies along two main dimensions. First, studies investigate their impact on access to high school. Empirical evidence shows that stricter policy thresholds significantly reduce the likelihood that migrant children enter high school, regardless of whether they migrate with their parents or remain behind. Policy simulations suggest that removing such restrictions could substantially increase high school enrollment among migrant children (Y. Y. Chen et al., 2023). Other studies using difference-in-differences approaches find that cross-regional enrollment policies can improve access to higher education, although these benefits are often concentrated among students from more advantaged family backgrounds (Song et al., 2025).

Second, the literature explores how cross-regional enrollment policies influence migration decisions. S. Wang et al. (2025) investigated the reform allowing migrant children to participate in the local Gaokao and found that expanding examination access significantly increased children’s migration to destination regions and reduced the probability of being left behind. Recent research using survey data shows that restrictive policies increase parents’ intentions to send children back to their place of origin, partly by lowering educational expectations and reducing investment in children’s education (B. Chen et al., 2026).

### 2.4. Institutional Constraints and Educational Inequality: The Role of Family Capital

The moderating role of family capital is another key issue in the literature (Ding & Wu, 2025). Empirical evidence shows a substantial gap in high school enrollment rates between migrant children and local students (Wei & Hou, 2010), reflecting the combined effects of institutional barriers and family resource constraints (Wu & Zhang, 2015).

Early studies, grounded in theories of institutional exclusion, emphasize the role of the household registration system in structuring unequal access to education (Zhou & Cheung, 2017). Building on this perspective, subsequent research examines how family socioeconomic status interacts with policy effects. Some studies find that the benefits of cross-regional enrollment policies are concentrated among families with more advantageous socioeconomic backgrounds (Song et al., 2025). Additional evidence suggests that stricter access thresholds have stronger negative effects on children from low-skilled or rural households (Y. Y. Chen et al., 2023).

These mixed findings point to an unresolved question: do cross-regional enrollment policies mitigate or reinforce educational inequality? From a behavioral perspective, this question hinges on how families with different resource endowments respond to institutional constraints, which remains underexplored.

### 2.5. Summary and Research Gap

In summary, the existing literature provides important insights into the institutional design and effects of cross-regional enrollment policies. However, three key gaps remain. First, most studies focus on migrant children who move with their parents, with limited consideration of left-behind children, potentially leading to sample selection bias. Second, the literature primarily examines school enrollment outcomes without distinguishing the location of schooling, which is central to evaluating policy effectiveness. Third, existing measures of policy stringency often rely on composite indices, with insufficient attention to the heterogeneous effects of specific eligibility requirements.

To address these gaps, this study adopts a behavioral perspective to examine how institutional constraints shape migrant families’ schooling decisions. It jointly considers migrant and left-behind children, distinguishes schooling location, and disaggregates policy conditions to identify their differential effects. In addition, it explores the role of family capital and the mechanism of parent–child co-migration, thereby providing a more comprehensive understanding of how policy design influences educational access and inequality. Based on the above analytical framework, this study proposes the following hypotheses:

**H1.**  *The implementation of cross-regional enrollment policies with more relaxed access thresholds significantly increases the likelihood that migrant children attend high school in destination areas.*

**H2.**  *Different types of access conditions—including residence permits, stable employment, stable housing, social security contributions, and school type restrictions—exert heterogeneous effects on migrant children’s educational opportunities.*

**H3.**  *Access conditions closely linked to family background exacerbate disparities in high school educational opportunities among migrant children from different socioeconomic backgrounds, thereby reinforcing inequality in the allocation of educational resources.*

**H4.**  *Cross-regional enrollment policies operate through the key channel of promoting early child co-migration, thereby reshaping household migration strategies.*

## 3. Materials and Methods

### 3.1. Data Sources

The data used in this study come from the China Migrants Dynamic Survey (CMDS), a nationwide survey conducted by the National Health Commission, which features a large sample size and broad geographic coverage, providing representative data. We utilized data from the 2012–2018 waves of the survey. To examine the effects of cross-regional enrollment policies, it is necessary to ensure that migrant families were already residing in the current destination cities when the children faced schooling decisions. Therefore, we retained only those households that had migrated to their current destination before the children reached high school age. It should be clarified that, in this study, “children from migrant families” refers to the full sample, while “migrant children” specifically denotes those who migrated with their parents, and “left-behind children” refers to those who remained in their place of origin.

### 3.2. Descriptive Statistics

We conducted descriptive statistical analyses on the population of children of high school age from migrant families in the database. We examined the patterns of high school attendance and migration before and after the implementation of cross-regional enrollment policies with strict and lenient access thresholds. Based on provincial policy documents and the existing literature (Li & Sun, 2025), Beijing, Shanghai, and Guangdong are classified as having stricter cross-regional enrollment policies, whereas Sichuan, Henan, Heilongjiang, Guangxi, Shaanxi, Hebei, Shanxi, Jiangxi, Anhui, Fujian, Zhejiang, Jiangsu, Liaoning, Shandong, Hunan, Hubei, and Chongqing are classified as having relatively lenient policies.

Regarding children from migrant families’ migration and high school attendance, four possible states were observed: migrated and attended high school, migrated and did not attend high school, left behind and attended high school, and left behind and did not attended high school. It is noteworthy that among children who migrated and attended high school, a small proportion had already partly accepted high school education prior to migration. But they were not admitted to high school through participation in the high school entrance examination in the destination areas and therefore did not benefit from the policy. Using information on migration timing and birth dates, we identified and separately reported this subgroup.

**Figure 1:**
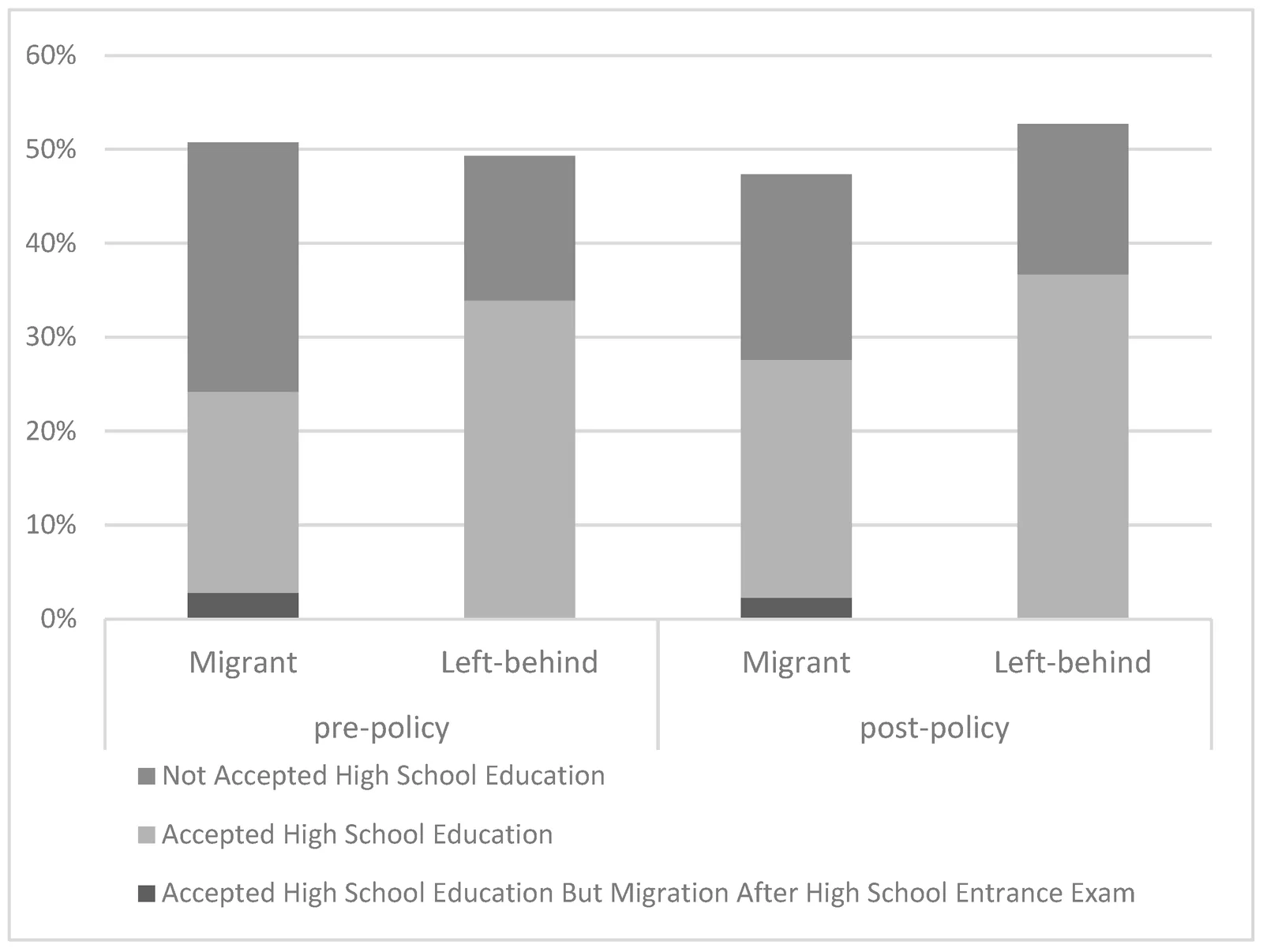
Descriptive Statistics before and after Implementation of Strict Access Thresholds.

Figure 1 shows the proportions of children in different migration and high school attendance states before and after the implementation of cross-regional enrollment policies in provinces with relatively strict access thresholds. Among migrant children, the proportion not attending high school was higher before policy implementation, whereas the proportion attending high school increased after policy implementation. This indicates that after the implementation of cross-regional enrollment policies, these provinces indeed opened up some high school opportunities for migrant children, increasing their chances of attending high school in the destination areas. For left-behind children, the proportion attending high school was higher both before and after policy implementation, with a slight increase following policy adoption. Overall, in provinces with strict access thresholds, left-behind children attending high school accounted for the largest share, and this share further increased after policy implementation. Despite the policy, a large number of migrant families in these provinces are still unable to both migrate with their children and secure high school education opportunities under strict eligibility conditions.

**Figure 2:**
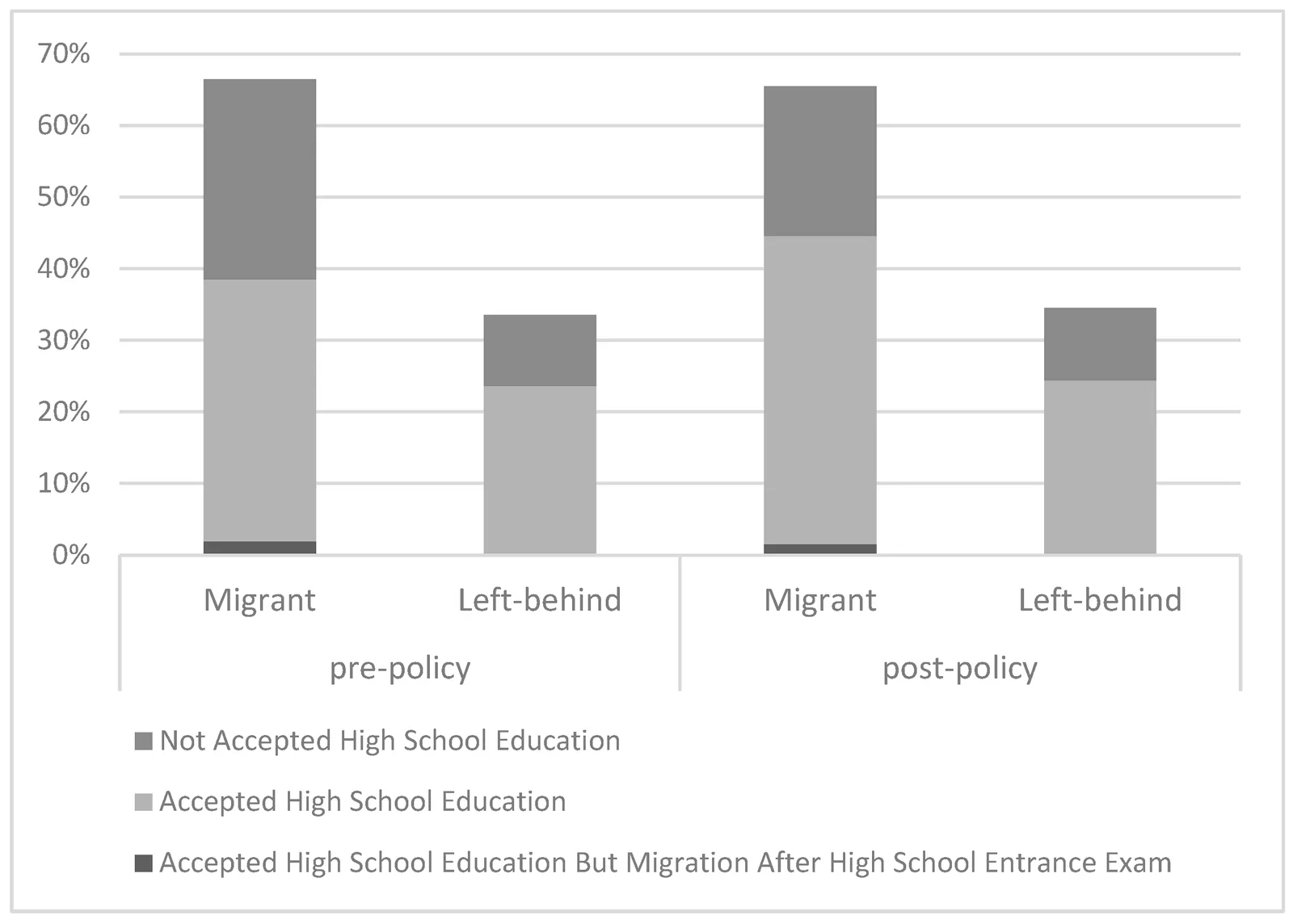
Descriptive Statistics before and after Implementation of Lenient Access Thresholds.

Figure 2 shows the proportions of children from migrant families in various migration and high school attendance states in provinces with lenient access thresholds. Both migrated and left-behind children experienced increases in high school attendance before and after policy implementation, with the growth among migrated children being more pronounced. Overall, in provinces with lenient access thresholds, migrated children attending high school accounted for the largest share, and this share increased further after policy implementation. Compared with provinces implementing strict access thresholds, provinces with lenient access thresholds allow a larger proportion of children from migrant families to simultaneously migrate with their families and attend high school.

In summary, by comparing Figure 1 and Figure 2, we find that in provinces with lenient cross-regional enrollment policies, both the migration rate and high school attendance opportunities for children from migrant families are higher, making it easier for them to attend high school in the destination cities. However, descriptive statistics cannot rule out the influence of other factors, so we subsequently construct an econometric model to investigate the causal effects of cross-regional enrollment policies.

### 3.3. Analytical Strategies: Variables, Models, and Statistical Procedures

To construct the variable “attending high school in the destination area,” we first identify which children from migrant families received high school education in their destination areas. The baseline regression sample consists of children from migrant families who are at high school age in each survey year. Based on their current place of residence and educational attainment, we first select those who reside in the destination areas and have received high school education. However, this group may include individuals who completed high school education prior to migration and then moved to their current residence. The same as the previous text, using information on migration timing and birth dates, we identify children who migrated with their families only after the typical time of high school enrollment and exclude them from the sample. The remaining individuals are those who received high school education in the destination areas. It should be noted that not all migrant children complete compulsory education on time, and delayed entry into high school may occur. We address this issue in the robustness checks.

To examine the impact of policy thresholds, provinces with relatively strict access thresholds are defined as the control group, while provinces with relatively lenient access thresholds are treated as the treatment group. Based on the timing of high school entry for each cohort and the timing of policy implementation, we divide the sample into pre-policy and post-policy cohorts. Cross-regional enrollment policies were introduced at the end of 2012 and gradually implemented during 2013–2014. In China, the academic year begins in September, and the typical age for high school attendance is 15–17. Accordingly, we divide all children into cohorts based on their birth dates, as shown in Table 1. Individuals born between September 1993 and August 1997 reached high school age in or before 2012 and were therefore not affected by the policy. In contrast, those born between September 1998 and August 2002 reached high school age in or after 2014 and were exposed to the policy. Cohorts highlighted on the left-hand side of Table 1 constitute the pre-policy group, while those highlighted on the right-hand side in bold constitute the post-policy group. Cohorts in the middle (italicized in the table) reached high school age during the transition period when the policy had been implemented in some provinces but not fully in others; thus, their exposure status is ambiguous, and they are excluded from the analysis.

Following previous studies (Duflo, 2001; Y. Chen et al., 2020a), we employ a cohort-based DID (Cohort DID) model to estimate the policy effects, as specified in Equation (1):

(1) $$Y_{i,p,c,t}=\beta_{0}+\beta_{1}{treat}_{p}+\beta_{2}{post}_{c}\left(199809\leq c\leq 200208\right)+\beta_{3}{treat}_{p}*{post}_{c}+\sum \Lambda_{k}X_{i,p,c,t}+\delta_{p}+\delta_{c}+\delta_{t}+\varepsilon_{i,p,c,t}$$

The dependent variable $Y_{i,p,c,t}$ indicates whether child i from a migrant family in cohort c , residing in province *p*, with migration timing t , has access to high school education and whether they received high school education in the destination area. If so, $Y_{i,p,c,t}$ is coded as 1; otherwise, it is 0. The variable ${treat}_{p}$ is a policy dummy based on the access thresholds of each province, representing policy intensity: provinces with stricter thresholds are coded as 0, and provinces with more relaxed thresholds are coded as 1. The variable ${post}_{c}$ is a cohort grouping variable, coded 0 for the pre-policy group and 1 for the post-policy group. The key independent variable of the model is the interaction term ${treat}_{p}*{post}_{c}$ , and its coefficient $\beta_{3}$ measures the effect of the access thresholds. A positive $\beta_{3}$ indicates that lenient access thresholds positively affect children from migrant families’ opportunities to attend high school in the destination area.

$X_{i,p,c,t}$ represents other control variables, including individual- and family-level characteristics. Individual-level variables include gender (male = 1, female = 0), whether the household has other minor children (yes = 1, no = 0), agricultural household registration (yes = 1, no = 0), ethnic minority (yes = 1, no = 0), migration scope (within-county = 1, cross-city within province = 2, cross-province = 3), and survey year. Family-level variables include parent’s maximum years of education and household per capita income. The parent’s maximum years of education are coded based on the highest educational attainment reported in the survey: “no schooling” = 0; primary school = 6; middle school = 9; high school/vocational school = 12; junior college = 15; bachelor = 16; postgraduate = 19. Regarding household economic status, two proxies are used. In the baseline regression, we use per capita income, calculated as the logarithm of the total monthly household income divided by household size. For robustness checks, considering that housing expenses are an important cost for migrant families, we use adjusted per capita income, calculated as the logarithm of (total monthly income minus average monthly housing expenditure) divided by household size.

Additionally, we control for cohort, migration timing and province fixed effects. To capture cohort fixed effect, the birth year and month of migrant children are coded as a single birth month variable. The migration timing fixed effect is the same as well. We employ linear probability models for estimation, with standard errors clustered at the provincial level to account for within-province correlation of errors. Descriptive statistics for the variables used in the regressions are reported in Table 2.

## 4. Results

### 4.1. Baseline Regression Results

Table 3 reports the baseline regression results. In the models, we sequentially include controls at different levels and fixed effects. Columns (1)–(3) use whether children from migrant families attended high school as the dependent variable, while columns (4)–(6) use whether children from migrant families attended high school in the destination area as the dependent variable.

The results in column (3) show that more relaxed cross-regional enrollment policies positively affect children from migrant families’ opportunities to attend high school, consistent with the existing literature (Y. Y. Chen et al., 2023). We further examine the policy effect on children from migrant families attending high school in the destination area. Column (4) reports the regression results controlling for individual- and family-level characteristics. The coefficient of the difference-in-differences term is positive and significant at the 5% level, indicating a significant positive association between the openness of cross-regional enrollment policies and migrant children’s access to high school education in the destination area.

To account for potential confounding factors, we sequentially include birth cohort, migration timing, and province fixed effects. The results show that the policy treatment effect remains statistically significant, confirming the robustness of our findings. The empirical evidence suggests that cross-regional enrollment policies significantly improve children from migrant families’ access to high school education in the destination area, alleviating household registration barriers to high school resources. Specifically, according to the full model in column (6), relative to strict threshold policies, the lenient cross-regional enrollment policies increase the probability of children from migrant families attending high school in the destination area by an average of 4.75 percentage points. Taken together, these results demonstrate that the implementation of cross-regional enrollment policies with more relaxed access thresholds significantly expands high school educational opportunities in destination areas for migrant children. Therefore, H1 is fully supported.

### 4.2. Test of the Parallel Trends Assumption

To ensure the validity of the difference-in-differences (DID) estimates, we conduct a test of the parallel trends assumption using an event-study approach, taking the earliest period as the reference group. This method allows us to visually and statistically examine whether there are significant differences in trends between the treatment and control groups prior to policy intervention, thereby providing empirical support for the applicability of the DID framework.

(2) $$Y_{i,p,c,t}=\beta_{0}+\sum_{j=1}^{n}\lambda_{j}{treat}_{i}\times T_{c}+\sum \Lambda_{k}X_{i,p,c,t}+\delta_{p}+\delta_{c}+\delta_{t}+\varepsilon_{i,p,c,t}$$

In this specification, $Y_{i,p,c,t}$ indicates whether children from migrant families attend high school and whether they attend high school in the destination area. $T_{c}$ denotes the birth cohort of migrant children, and its interaction with the treatment indicator is included in the regression model. If the coefficients on all interaction terms for the pre-policy periods are statistically insignificant, this suggests that the treatment and control groups exhibit a credible parallel pre-trend, thereby supporting the causal identification of the DID strategy.

**Figure 3:**
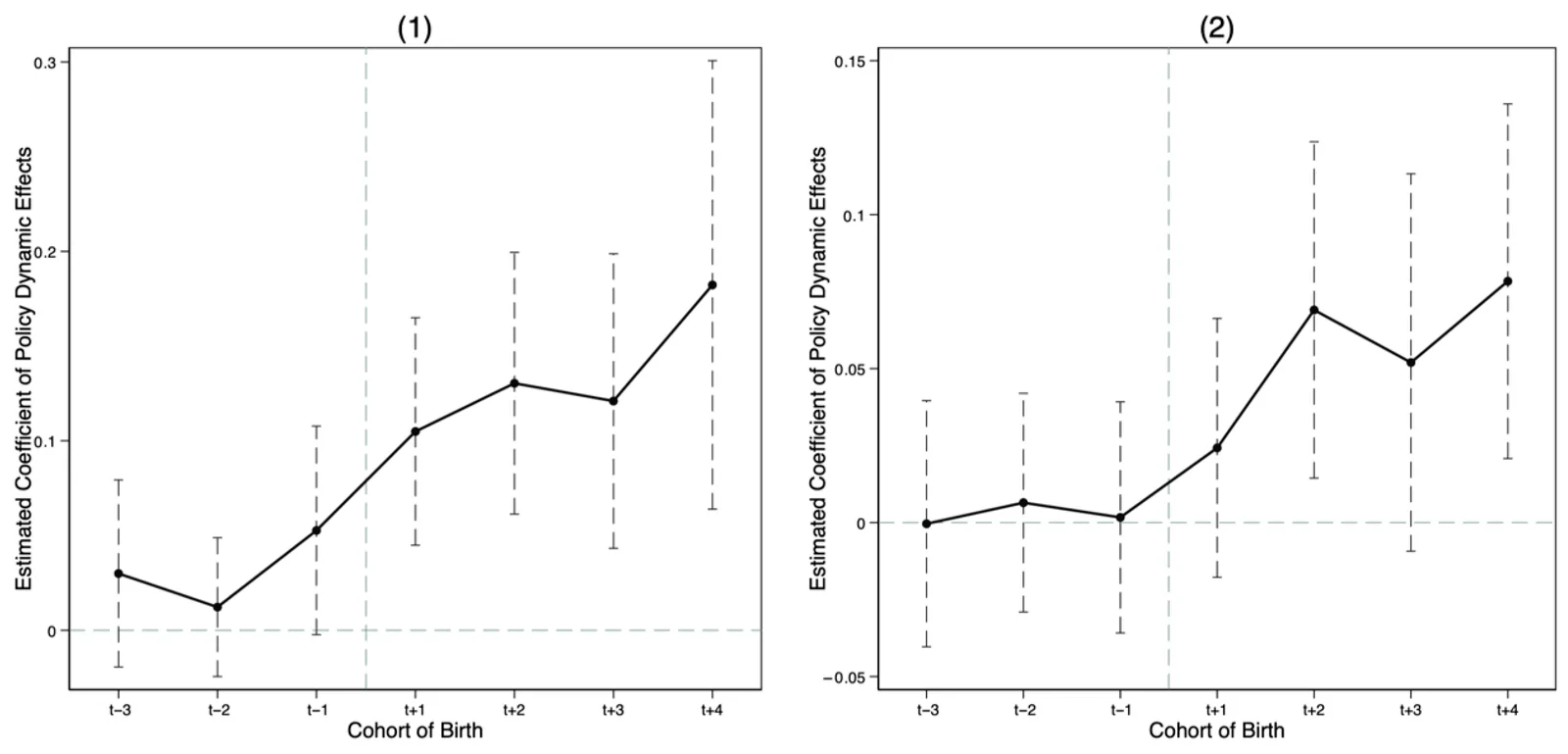
(**1**) Parallel Trends Assumption Test Results for Whether Attended High School; (**2**) Parallel Trends Assumption Test Results for Whether Attended High School in Destination Area.

The results are presented in Figure 3. All estimated pre-policy effects are statistically insignificant, indicating that the parallel trends assumption is satisfied. Moreover, the direction of the coefficients is consistent with that of the DID estimates in the baseline regressions.

### 4.3. Placebo Tests

To further enhance the credibility of the identification strategy and rule out potential confounding factors, we conduct both time placebo tests and individual placebo tests to verify that the observed treatment effects are indeed driven by the implementation of cross-regional enrollment policies. First, we artificially shift the policy implementation time forward to construct a false policy onset and redefine the pre- and post-policy groups accordingly. This allows us to examine whether significant “treatment effects” would be observed in periods when the policy had not yet been implemented. If the estimated coefficients are insignificant under this specification, it suggests that the identified policy effects are not driven by underlying time trends or other exogenous factors.

Since the implementation of the policy varied across provinces (with some provinces implementing it in 2013 and others following in 2014), we avoid overlap with the actual policy timing by setting placebo policy dates starting two years earlier and moving backward year by year. Time placebo tests are conducted for both baseline regressions, and the results are reported in Table 4. The coefficients on the DID interaction term are statistically insignificant across all specifications, indicating that the time placebo tests are passed, and strengthen the credibility of the empirical findings.

**Figure 4:**
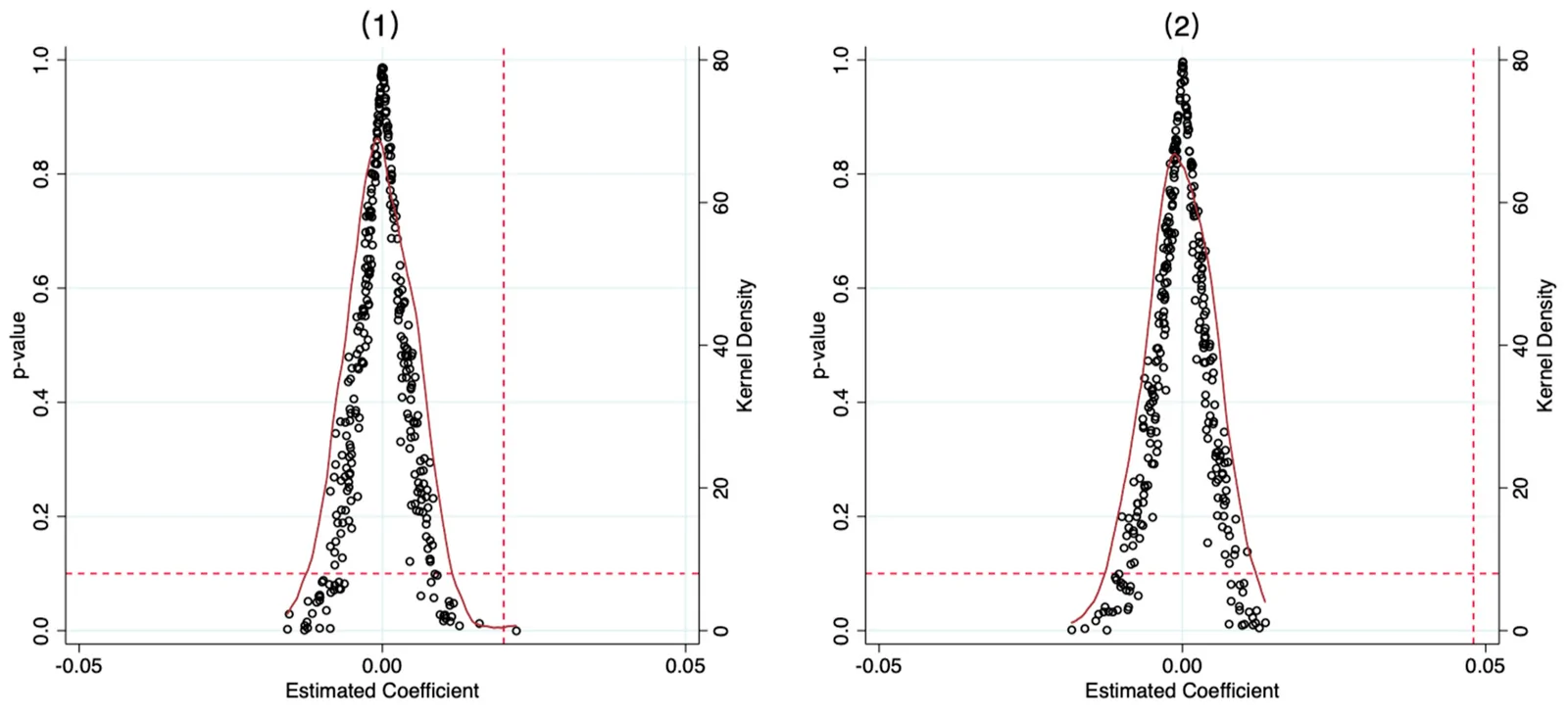
(**1**) Individual Placebo Test Results for Whether Attended High School; (**2**) Individual Placebo Test Results for Whether Attended High School in Destination Area.

Next, we conduct an individual placebo test. Specifically, we randomly assign the treatment group and repeat the regression 300 times. The distribution of the estimated coefficients is presented in Figure 4.

As shown in the figure, most coefficients are concentrated around zero and are statistically insignificant. Moreover, the distribution is far from the baseline DID estimate, indicating that the results are unlikely to be driven by random variation or omitted variables. Therefore, the individual placebo test is also passed.

### 4.4. Robustness Checks

#### 4.4.1. Effects of Different Eligibility Requirements

To examine the robustness of the baseline regression results, we conduct a series of robustness checks. We further investigate how different types of eligibility requirements in cross-regional enrollment policies affect children from migrant families’ access to high school education in the destination area. Provincial policies set separate requirements for the cross-regional high school entrance examination and the cross-regional college entrance examination. In this study, we focus on the eligibility conditions for the latter as the benchmark.

Except for Shanghai, which adopts a points-based system, the eligibility requirements for the cross-regional college entrance examination in other provinces can be categorized into four types: residence permits, stable employment, stable housing, and social security contributions (including tax payments and pension insurance). In addition, Beijing imposes restrictions on the types of high schools that migrant children are allowed to apply for.

To examine the effects of each type of requirement, we replace the DID interaction term in the baseline regression with interaction terms between each policy-specific dummy variable and the cohort grouping variable, while keeping other specifications unchanged. In this setting, ${treat}_{p}$ indicates whether province p imposes a given requirement (1 = yes, 0 = no). The coefficient $\beta_{3}$ captures the effect of the requirement. A negative $\beta_{3}$ indicates that the requirement reduces children from migrant families’ likelihood of attending high school in the destination area. The results are reported in Table 5.

The results show that residence permit requirements and school type restrictions have the largest and most statistically significant negative effects. A comparison of coefficient magnitudes indicates that restrictions on school types impose the strongest negative effect, followed by residence permit requirements. The results confirm that only administrative and school-type constraints significantly restrict enrollment, while socio-economic criteria do not, demonstrating clear heterogeneity. Therefore, H2 is fully supported.

#### 4.4.2. Additional Robustness Checks

We conduct several additional robustness checks. First, some migrant families move primarily to access better educational resources for their children. Including such observations may introduce sample selection bias. Therefore, we exclude households whose migration purpose is education and re-estimate the model. Second, China implemented the “two priorities” policy in 2001 to address compulsory education for migrant children, which may have long-term effects on subsequent educational attainment. To isolate the impact of other policies, we exclude children who entered primary school before 2000. Third, given that housing expenditures constitute a substantial share of household expenses for migrant families, we construct an alternative measure of household income by subtracting housing costs from total income and re-estimate the model using this adjusted per capita income variable. Fourth, delayed school enrollment may occur among children from migrant families, especially those from disadvantaged backgrounds. As a result, some individuals classified as being of Grade 10 age in the baseline sample may still be in lower grades and have not yet taken the high school entrance examination. To address this concern, we construct a subsample excluding Grade 10-age children and re-estimate the model. The regression results are reported in Table 6. The coefficients on the DID interaction term remain positive and statistically significant across all specifications, consistent with the baseline results.

### 4.5. Heterogeneity Analysis

Since cross-regional enrollment policies may affect children from migrant families differently depending on their family capital, we conduct a heterogeneity analysis to examine the effects of these policies on migrant children’s opportunities to attend high school in the destination area. We also compare the effects of strict versus lenient enrollment policies.

The design of access thresholds in cross-regional enrollment policies is closely related to family background. It can be anticipated that children from families with higher economic or educational capital are more likely to meet these thresholds, thereby gaining access to high school education in the destination area. If true, family capital could perpetuate intergenerational inequality in education, hindering educational equity. To examine the heterogeneous effects of family capital, we construct a model as follows:

(3) $$Y_{i,p,c,t}=\beta_{0}+\beta_{1}{capital}_{i,p,c,t}+\beta_{2}{post}_{c}\left(199809\leq c\leq 200208\right)+\beta_{3}{capital}_{i,p,c,t}*{post}_{c}+\sum \Lambda_{k}X_{i,p,c,t}+\delta_{p}+\delta_{c}+\delta_{t}+\varepsilon_{i,p,c,t}$$

Here, $Y_{i,p,c,t}$ is a binary outcome indicating whether child i has the opportunity to attend high school in the destination area (1 = yes, 0 = no). ${capital}_{i,p,c,t}$ represents family capital, proxied by household per capita income for economic capital and highest years of education among adult family members for educational capital. The key variable is the interaction ${capital}_{i,p,c,t}*{post}_{c}$ , whose coefficient $\beta_{3}$ captures the heterogeneous effect of the policy across different levels of family capital. A positive $\beta_{3}$ indicates that children from families with higher capital gain more high school access in the destination area following policy implementation (Table 7).

The results indicate that for economic capital, overall and lenient-threshold policies show no significant effects, while strict-threshold policies benefit children from higher-income families more. For cultural capital, children from lower-education households experience larger positive effects, especially under strict-threshold policies. These findings suggest that cross-regional enrollment policies increase disparities in access by economic capital while reducing disparities by cultural capital. While we hypothesized that access conditions linked to family background would widen disparities across all dimensions, our empirical results reveal significant heterogeneity: the hypothesis is supported regarding the economic capital dimension, but not supported regarding the cultural capital dimension. Therefore, H3 is partially supported. The findings on economic capital are relatively straightforward to interpret. Since the eligibility requirements of cross-regional enrollment policies are closely tied to family conditions, children from migrant families with higher levels of economic capital are more likely to meet these requirements after policy implementation, thereby gaining greater access to educational opportunities in the destination area.

Regarding cultural capital, existing studies have shown that there is a substantial disparity in access to education in the destination area between migrant children from high-cultural-capital families and those from low-cultural-capital families, and that educational opportunities for those from high-cultural-capital families are already close to saturation (Li & Sun, 2025). One possible explanation is that, prior to the implementation of the policy, children from migrant families with lower levels of cultural capital faced more severe constraints in educational access, leaving greater room for improvement after the policy was introduced. In contrast, migrant children from families with higher cultural capital already have high rates of high school enrollment, and thus experience smaller marginal gains. This pattern is consistent with the Maximally Maintained Inequality (MMI) hypothesis (Raftery & Hout, 1993), which posits that when the educational demands of higher-status groups are largely satisfied, newly expanded opportunities are more likely to benefit lower-status groups.

### 4.6. Mechanism Analysis

The impact of educational barriers extends far beyond the transition to higher levels of schooling. A more fundamental and upstream issue lies in family migration decisions. As a critical stage linking compulsory education to higher education or the labor market, the policy environment surrounding high school education plays a crucial role in shaping both household migration decisions and children’s developmental trajectories.

Importantly, this influence is not confined to the point of high school entry but may occur earlier, during the junior high school stage. More inclusive high school entrance policies may incentivize migrant families to arrange for their children to migrate at an earlier stage, thereby laying the foundation for subsequent high school enrollment in destination areas. To examine this mechanism, we investigate the impact of cross-regional enrollment policies on children’s co-migration decisions within migrant families, focusing on this upstream channel. Specifically, we conduct separate regressions for children of high school age and junior high school age, based on the following cohort-based difference-in-differences model:

(4) $$Y_{i,p,c,t}=\beta_{0}+\beta_{1}{treat}_{p}+\beta_{2}{post}_{c}\left(199809\leq c\leq 200208\right)+\beta_{3}{treat}_{p}*{post}_{c}+\sum \Lambda_{k}X_{i,p,c,t}+\delta_{p}+\delta_{c}+\delta_{t}+\varepsilon_{i,p,c,t}$$

where $Y_{i,p,c,t}$ is a binary variable indicating whether a child migrates with the family. The variable ${treat}_{p}$ is defined as in the baseline regression and represents policy intensity, with provinces characterized by stricter eligibility requirements coded as 0 and those with more lenient policies coded as 1. The variable ${post}_{c}$ is a cohort indicator, taking the value of 1 for post-policy cohorts and 0 otherwise. The interaction term ${treat}_{p}*{post}_{c}$ is the key explanatory variable, and the coefficient $\beta_{3}$ captures the policy effect. A positive $\beta_{3}$ indicates that more lenient policies increase the likelihood of children migrating with their families.

The results in Table 8 reveal substantial heterogeneity in policy effects across age groups, highlighting the underlying mechanism. For children at the high school stage, more lenient cross-regional enrollment policies increase the probability of co-migration by 4.68 percentage points, indicating that policy improvements directly facilitate migration decisions at the stage. More strikingly, the policy exerts a much stronger effect on children at the junior high school stage, increasing the likelihood of co-migration by 20.3 percentage points. This substantial difference suggests a clear upstream effect of the policy: more favorable entrance policies incentivize migrant families to bring their children along at an earlier stage, thereby securing access to local high school education in advance.

Overall, these findings provide strong evidence that children’s co-migration serves as a key mediating channel through which the policy operates. Therefore, H4 is fully supported. By shifting migration decisions forward in time, the policy effectively reduces future educational barriers and improves access to high school education in destination areas.

## 5. Discussion

This study examines how the institutional constraints embedded in cross-regional enrollment policies shape educational decisions among migrant families in China. The findings provide multi-level evidence on how individuals and households respond behaviorally to policy-induced constraints in the context of education, population mobility, and the formation of social belonging, offering empirical insights for building a more inclusive and sustainable educational governance system in which institutional design is closely linked to identity-related outcomes. To provide a comprehensive overview of the empirical evidence gathered in this study, Table 9 summarizes the testing results and the level of support for each hypothesis, which are discussed in detail below.

First, the results demonstrate that institutional design plays a significant role in shaping behavioral responses to educational access policies. Consistent with the existing literature, cross-regional enrollment policies increase the likelihood that migrant children attend senior secondary education in the destination area; however, this effect is significantly weakened under stricter access thresholds (Y. Y. Chen et al., 2023). This indicates that policy effectiveness depends not only on formally granted educational rights, but also on the behavioral constraints embedded in implementation rules (Ma & Wang, 2025; Y. Chen et al., 2020b). From the perspective of sustainable educational development, institutional design also indirectly shapes the conditions under which migrant families can establish a sense of educational belonging in destination cities. Only by incorporating behavioral responses and inclusion dynamics into policy evaluation frameworks can the institutional sustainability of educational opportunity provision be ensured.

Second, the study reveals substantial heterogeneity in household responses to different types of institutional constraints. Among various eligibility conditions, eligibility requirements related to household registration status and school type emerge as the most binding constraints. This suggests that not all access standards exert equivalent behavioral pressure (L. Wang, 2016). More rigid and resource-dependent requirements are more likely to exclude disadvantaged families from both educational opportunities and the institutional pathways through which social membership in destination areas is constructed. This finding implies that achieving sustainable educational equity requires identifying and prioritizing the relaxation of those “high-binding, high-exclusion” institutional barriers in order to reduce the cumulative exclusionary effects of structural constraints on both opportunities and social integration.

Third, the results contribute to the literature on inequality by highlighting the interaction between institutional constraints and household resources, which generates stratified behavioral outcomes and differentiated patterns of social positioning. Consistent with prior studies, families with higher economic capital are more capable of meeting eligibility conditions and thus more likely to access schooling in the destination area, whereas resource-constrained households face stronger institutional barriers and more limited behavioral flexibility (Bo & Wang, 2025; Ding & Wu, 2025). Such heterogeneous responses to the same institutional environment further exacerbate existing inequalities (Song et al., 2025). The analysis of cultural capital also supports the Maximally Maintained Inequality (MMI) theory (Raftery & Hout, 1993), suggesting that when educational demand among advantaged groups reaches saturation, newly expanded opportunities tend to be allocated to lower-status groups. From the perspective of social sustainability, these stratification processes also imply differentiated trajectories of social identity formation, where access to education becomes closely tied to unequal forms of institutional belonging. If policy design does not actively address resource-driven stratification mechanisms, it may unintentionally reproduce or reinforce both class structures and differentiated identity boundaries within urban education systems.

Fourth, the findings underscore the importance of trade-offs in household decision-making. When confronted with binding institutional constraints, migrant families are often forced to choose between accessing educational opportunities in the destination area and maintaining family co-residence (S. Wang et al., 2025). This trade-off reflects a form of bounded rational optimization under institutional and economic constraints, whereby educational decisions are jointly determined by policy eligibility conditions and household utility considerations (B. Chen et al., 2026). Evidence from the analysis of parent–child co-migration further confirms that migration decisions and educational choices are interdependent rather than independent processes, highlighting intra-household behavioral interdependence. These findings suggest that lenient policies trigger an anticipatory migration channel, where families engage in forward-looking, long-term educational planning during the compulsory schooling stage. This upstream family co-migration may potentially shape parental labor market dynamics. Bringing junior high school children along early tends to anchor the parents’ labor supply in the destination city. It could shift their behavioral logic from transient, circular migration toward permanent urban settlement, incentivizing parents to transition from informal, short-term jobs to stable, long-term employment.

Finally, the study emphasizes broader policy implications. Without accounting for behavioral responses to institutional design, it is difficult to fully understand educational outcomes and their implications for social integration. To achieve institutional sustainability in educational access and urban integration, policymakers need to move beyond average treatment effect evaluations and systematically identify heterogeneous behavioral responses across different household groups, particularly those that reflect unequal conditions of social belonging. Based on these insights, a more inclusive, adaptive, and dynamically adjustable system of cross-regional enrollment policies should be developed (Yu & Crowley, 2020). Only by embedding behavioral equity and inclusion-sensitive institutional design into the core of policy evaluation can a more just, resilient, and socially integrative trajectory of educational development be achieved in the context of increasing population mobility in urban China.

## 6. Conclusions

This study examines how institutional constraints embedded in cross-regional enrollment policies shape migrant families’ schooling decisions in China. Using data from the China Migrants Dynamic Survey (2012–2018) and a cohort-based difference-in-differences (DID) approach, we analyze the effects of eligibility thresholds on high school enrollment and schooling location choices. The results show that cross-regional enrollment policies with stricter eligibility requirements significantly weaken the likelihood that migrant children attend high school in destination areas, indicating that institutional barriers play a decisive role not only in shaping educational access but also in structuring the conditions under which migrant families can establish stable social belonging in destination cities. When facing binding constraints, migrant families are more likely to experience trade-offs between educational opportunities and family co-residence, reflecting how institutional design shapes the lived experience and negotiation of migrant social identity.

Further analysis reveals substantial heterogeneity across different types of eligibility conditions. Residency requirements and school-type restrictions emerge as the most influential constraints on schooling decisions, effectively defining the boundaries of institutional inclusion. In addition, these institutional barriers amplify the role of household economic capital in determining access to educational opportunities, while partially mitigating disparities associated with cultural capital. Mechanism analysis further highlights parent–child co-migration as a key behavioral channel through which institutional constraints operate. Beyond its role in shaping educational trajectories, co-migration also determines the spatial and relational contexts in which migrant children’s social identities are formed and experienced.

A conceptual limitation regarding the measurement of social identity should be noted. While our theoretical framework emphasizes “social identity formation,” the empirical variables primarily capture educational and mobility behaviors. Social identity is thus operationalized through the lens of structural inclusion and institutional membership, rather than directly measured via psychometric instruments or attitudinal surveys. Future research combining behavioral econometric analysis with psychological scales would provide a more complete picture of how these institutional barriers translate into internalized subjective identities.

Overall, the findings suggest that migrant families’ schooling decisions are highly responsive to institutional design. More importantly, educational access under cross-regional enrollment policies is jointly shaped by policy constraints, family resources, and migration behavior, and these factors together contribute to the production of differentiated patterns of social belonging and identity formation among migrant children.

## 7. Implications

From a behavioral and policy design perspective, this study highlights several implications for improving the equity and effectiveness of cross-regional enrollment policies, particularly in relation to how institutional arrangements shape migrant families’ access to educational opportunities and their broader experiences of social belonging in destination cities. First, reducing excessive eligibility thresholds is essential for enhancing both the effectiveness of cross-regional enrollment policies and the inclusiveness of migrant families’ institutional membership. While conditional access mechanisms may help manage local educational capacity, overly strict requirements significantly constrain migrant families’ schooling decisions and limit the formation of stable educational belonging in destination areas. More flexible admission criteria would allow families to make educational choices with fewer institutional distortions and support more inclusive pathways of integration.

Second, policy design should differentiate between types of institutional constraints, as their effects on behavior and inclusion are not uniform. The findings show that residency-based requirements and school-type restrictions impose the strongest constraints on migrant families’ educational access and, by extension, their sense of institutional inclusion. Policymakers should therefore prioritize simplifying administrative procedures and reducing rigid residency-linked barriers, while maintaining reasonable safeguards for resource allocation. Such reforms would help reduce the segmentation of institutional membership across population groups.

Third, attention should be given to the unequal behavioral responses induced by institutional constraints. Eligibility conditions that are closely tied to family socioeconomic resources tend to reinforce pre-existing inequalities in educational access and contribute to the stratification of opportunity structures. Critically, the claim that reducing institutional barriers can mitigate disparities in cultural capital provides strong empirical support for the Maximally Maintained Inequality (MMI) hypothesis. From a social sustainability and identity perspective, adopting more inclusive policy designs is crucial to prevent institutional barriers from reproducing and amplifying intergenerational inequality, thereby ensuring that access to education does not become a mechanism of differentiated social belonging.

Fourth, the role of parent–child co-migration suggests that education policy and migration behavior are deeply interdependent processes. Restrictions on access to schooling in destination areas may unintentionally shape family migration strategies, including decisions regarding co-residence and separation. These behavioral adjustments not only affect educational trajectories but also influence the spatial and relational contexts in which migrant children’s social identities are formed. Therefore, improving educational access may also contribute to more stable, family-centered migration patterns and more coherent identity development processes. Overall, policy reforms should move toward a more inclusive and flexible institutional framework that reduces unnecessary constraints, aligns educational access with family needs, and supports more balanced outcomes between resource allocation efficiency, educational equity, and the formation of inclusive social identities among migrant populations.

## Figures and Tables

**Table 1:** Cohorts by Survey Year and Group Classification.

Survey Year	2012	2013	2014	2015	2016	2017	2018
Grade 12	1993.09–1994.08	1994.09–1997.08	1995.09–1996.08	1996.09–1997.08	*1997.09–1998.08*	**1998.09–1999.08**	**1999.09–2000.08**
Grade 11	1994.09–1995.08	1995.09–1996.08	1996.09–1997.08	*1997.09–1998.08*	**1998.09–1999.08**	**1999.09–2000.08**	**2000.09–2001.08**
Grade 10	1995.09–1996.08	1996.09–1997.08	*1997.09–1998.08*	**1998.09–1999.08**	**1999.09–2000.08**	**2000.09–2001.08**	**2001.09–2002.08**

Note: Cohorts highlighted on the left-hand side of Table 1 constitute the pre-policy group, while those highlighted on the right-hand side in bold constitute the post-policy group. Cohorts in the middle (italicized in the table) reached high school age during the transition period when the policy had been implemented in some provinces but not fully in others; thus, their exposure status is ambiguous, and they are excluded from the analysis.

**Table 2:** Descriptive Statistics of Variables.

Variable Category	Variable	Observations	Mean	Std. Dev.
Dependent Variables	Attended high school (yes = 1, no = 0)	58,861	0.643	0.479
	Attended high school in destination area (yes = 1, no = 0)	58,861	0.362	0.481
Key Independent Variables	${post}_{c}$ (pre-policy = 0, post-policy = 1)	58,861	0.443	0.497
	${treat}_{p}$ (Strict Threshold = 0, Lenient Threshold = 1)	58,861	0.801	0.399
Control Variables	Gender (male = 1, female = 0)	58,861	0.561	0.496
	Other minor children in household (yes = 1, no = 0)	58,861	0.480	0.500
	Agricultural household registration (yes = 1, no = 0)	58,861	0.862	0.345
	Ethnic minority (yes = 1, no = 0)	58,861	0.048	0.213
	Migration scope (within-county = 1, cross-city = 2, cross-province = 3)	58,861	2.327	0.773
	Survey year	58,861	2014.7	2.064
	Father’s maximum years of education	58,861	10.73	2.805
	Log of household per capita income	58,861	8.878	0.298
Fixed Effects	Cohort, migration timing, province			

**Table 3:** Effects of cross-regional enrollment policies on children from migrant families’ high school attendance.

	(1)	(2)	(3)	(4)	(5)	(6)
Dependent Variable	Attended High School	Attended High School	Attended High School	Attended High School in Destination Area	Attended High School in Destination Area	Attended High School in Destination Area
${treat}_{p}*{post}_{c}$	0.0291	0.0314	0.0192 *	0.0823 **	0.117 ***	0.0475 **
	(0.0235)	(0.0296)	(0.00971)	(0.0299)	(0.0367)	(0.0192)
Individual Variables	Yes	Yes	Yes	Yes	Yes	Yes
Family Variables	Yes	Yes	Yes	Yes	Yes	Yes
Birth Cohort FE		Yes	Yes		Yes	Yes
Migration Timing FE		Yes	Yes		Yes	Yes
Province FE			Yes			Yes
Observations	58,861	58,861	58,861	58,861	58,861	58,861
R-squared	0.228	0.242	0.254	0.135	0.162	0.194

Note: Standard errors in parentheses; *** *p* < 0.01, ** *p* < 0.05, * *p* < 0.1; FE = Fixed Effects.

**Table 4:** Time Placebo Test Results.

	(1)	(2)	(3)	(4)
Lead Time	Two Years Earlier	Three Years Earlier	Two Years Earlier	Three Years Earlier
Dependent Variable	Attended High School		Attended High School in Destination Area	
${treat}_{p}*{post}_{c}$	0.0138	0.0185	0.0237	0.0224
	(0.0109)	(0.0302)	(0.0150)	(0.0164)
Other Controls	Yes	Yes	Yes	Yes
FE	Yes	Yes	Yes	Yes
Observations	58,909	62,924	58,909	62,924
R-squared	0.246	0.223	0.188	0.184

Note: Standard errors in parentheses; FE = Fixed Effects.

**Table 5:** Effects of Different Eligibility Requirements on High School Attendance in the Destination Area.

	(1)	(2)	(3)	(4)	(5)
Requirement Type	Residence Permit	Stable Employment	Stable Housing	Social Security Contributions	School Type Restrictions
Dependent Variable	Attended High School in Destination Area				
${treat}_{p}*{post}_{c}$	−0.0322 *	−0.0137	−0.00377	−0.0195	−0.0738 ***
	(0.0164)	(0.0183)	(0.0176)	(0.0193)	(0.00973)
Other Controls	Yes	Yes	Yes	Yes	Yes
FE	Yes	Yes	Yes	Yes	Yes
Observations	50,492	50,492	50,492	50,492	50,492
R-squared	0.199	0.199	0.198	0.199	0.199

Note: Standard errors in parentheses; *** *p* < 0.01, * *p* < 0.1; FE = Fixed Effects.

**Table 6:** Robustness Check Results.

	(1)	(2)	(3)	(4)
	Sample Selection Adjustment	Excluding Other Policies	Accounting for Housing Costs	Accounting for Delayed Enrollment
Dependent Variable	Attended High School in Destination Area			
${treat}_{p}*{post}_{c}$	0.0433 *	0.0496 *	0.0466 **	0.0444 **
	(0.0231)	(0.0239)	(0.0189)	(0.0203)
Other Controls	Yes	Yes	Yes	Yes
FE	Yes	Yes	Yes	Yes
Observations	44,762	48,961	58,656	56,115
R-squared	0.213	0.187	0.194	0.196

Note: Standard errors in parentheses; ** *p* < 0.05, * *p* < 0.1; FE = Fixed Effects.

**Table 7:** Heterogeneity Analysis by Family Capital.

	(1)	(2)	(3)	(4)	(5)	(6)
	Economic Capital			Cultural Capital		
	Overall Policy	Strict Threshold	Lenient Threshold	Overall Policy	Strict Threshold	Lenient Threshold
Dependent Variable	Attended High School in Destination Area					
${capital}_{i,p,c,t}*{post}_{c}$	0.0350	0.0380 *	0.0375	−0.0138 ***	−0.0142 ***	−0.00897 *
	(0.0206)	(0.0215)	(0.0379)	(0.00339)	(0.00447)	(0.00222)
Other Controls	Yes	Yes	Yes	Yes	Yes	Yes
FE	Yes	Yes	Yes	Yes	Yes	Yes
Observations	58,861	47,193	11,668	58,861	47,193	11,668
R-squared	0.194	0.190	0.192	0.195	0.191	0.193

Note: Standard errors in parentheses; *** *p* < 0.01, * *p* < 0.1; FE = Fixed Effects.

**Table 8:** Effects of Cross-Regional High School Entrance Policy on Children’s Co-Migration with Families.

	(1)	(2)
	High School–Age Children	Junior High School–Age Children
Dependent Variable	Co-Migration	Co-Migration
${treat}_{p}*{post}_{c}$	0.0468 ***	0.203 ***
	(0.0101)	(0.0342)
Other Controls	Yes	Yes
FE	Yes	Yes
Observations	58,861	56,744
R-squared	0.124	0.226

Note: Standard errors in parentheses; *** *p* < 0.01; FE = Fixed Effects.

**Table 9:** Summary of Hypothesis Testing Results.

Hypothesis	Core Theoretical Statement	Support Status/Decision
H1	The implementation of cross-regional enrollment policies with more relaxed access thresholds significantly increases the likelihood that migrant children attend high school in destination areas.	Fully Supported
H2	Different types of access conditions (residence permits, stable employment, housing, social security, and school type restrictions) exert heterogeneous effects on migrant children’s educational opportunities.	Fully Supported
H3	Access conditions linked to family background exacerbate disparities in high school educational opportunities among migrant children from different socioeconomic backgrounds.	Partially Supported (Supported for economic capital; unsupported for cultural capital)
H4	Cross-regional enrollment policies operate through the key channel of promoting early child co-migration, thereby reshaping household migration strategies.	Fully Supported

## Data Availability

The data presented in this study are available on request from the corresponding author. The data are not publicly available due to privacy restrictions.
