# Correction: Li et al. (2026). Institutional Barriers, Educational Decisions, and the Forging of Migrant Social Identity: Evidence from China’s Cross-Regional Enrollment Policies. *Behavioral Sciences*, *16*(9), 1488

**DOI:** 10.3390/bs16111967

**Published:** 2026-10-22

**Authors:** Chen Li, Qiao Wen, Xu Li

**Affiliations:** 1 Business School, Beijing Normal University, Beijing 100875, China; 2 Education Development Research Center, Beijing Academy of Educational Sciences, Beijing 100036, China

## Error in Table

In the original publication (Li et al., 2026), there was a mistake in Table 3 as published. Authors identified a minor data entry error in the last row of Table 3 (i.e., R-squared). The corrected Table 3 appears below. The authors state that the scientific conclusions are unaffected. This correction was approved by the Academic Editor. The original publication has also been updated.

## Figures and Tables

**Table 3:** Effects of cross-regional enrollment policies on children from migrant families’ high school attendance.

	(1)	(2)	(3)	(4)	(5)	(6)
Dependent Variable	Attended High School	Attended High School	Attended High School	Attended High School in Destination Area	Attended High School in Destination Area	Attended High School in Destination Area
${treat}_{p}*{post}_{c}$	0.0291	0.0314	0.0192 *	0.0823 **	0.117 ***	0.0475 **
	(0.0235)	(0.0296)	(0.00971)	(0.0299)	(0.0367)	(0.0192)
Individual Variables	Yes	Yes	Yes	Yes	Yes	Yes
Family Variables	Yes	Yes	Yes	Yes	Yes	Yes
Birth Cohort FE		Yes	Yes		Yes	Yes
Migration Timing FE		Yes	Yes		Yes	Yes
Province FE			Yes			Yes
Observations	58,861	58,861	58,861	58,861	58,861	58,861
R-squared	0.228	0.242	0.254	0.135	0.162	0.194

Note: Standard errors in parentheses; *** *p* < 0.01, ** *p* < 0.05, * *p* < 0.1; FE = Fixed Effects.
